# Supplementary material for: Morphological characterization and staging of bumble bee pupae
Source: PeerJ. 2018 Dec 18;6:e6089. doi: 10.7717/peerj.6089 (PMC6302898; doi:10.7717/peerj.6089)
Supplement: Supplemental Information 3 — Numbers in the parenthesis indicates number of colonies used. [file peerj-06-6089-s003.docx]

**Supplemental tables and figures**

| Groups | 32°C | | | 29°C | | |
| --- | --- | --- | --- | --- | --- | --- |
|  | N | Mean Duration (Hours) | SE | N | Mean Duration  (Hours) | SE |
| Small worker | 18 (2) | 161.9 | 1.5 | 7 (1) | 181.0 | 1.7 |
| Medium worker | 20 (4) | 172.2 | 1.4 | 6 (2) | 206.0 | 1.8 |
| Large worker | 15 (4) | 179.6 | 1.6 | 6 (2) | 207.3 | 1.8 |
| Small male | 7 (2) | 188.6 | 2.4 | 3 (2) | 203.6 | 2.6 |
| Medium male | 13 (3) | 189.4 | 1.7 |  |  |  |
| Queen | 8 (1) | 243.2 | 2.2 | 8 (1) | 265.0 | 1.6 |
| Small male (*B.vosnesenskii*) | 8 (1) | 178.6 | 1.8 | 3(1) | 203.8 | 3.9 |

**Supplemental Table S1.** Descriptive statistics for pupal duration. Numbers in the parenthesis indicates number of colonies used.
