# Supplementary material for: Morphological characterization and staging of bumble bee pupae
Source: PeerJ. 2018 Dec 18;6:e6089. doi: 10.7717/peerj.6089 (PMC6302898; doi:10.7717/peerj.6089)
Supplement: Supplemental Information 4 — aA. mellifera staging based on Rembold, Kremer & Ulrich (1980); bData is represented as 95% confidence interval for medium sized workers. [file peerj-06-6089-s004.docx]

| *A. mellifera^a^* | | | *B. impatiens* | |
| --- | --- | --- | --- | --- |
| Stage | Definition | Duration  (hours) | Stage | Duration  (hours)^b^ |
| Pw | White eyed | 30-42 | P0 | 0.1-4 |
|  |  |  | P1 | 5-8 |
|  |  |  | P2 | 10-16 |
| Pp | Pink eyed | 18-30 | P3 | 10-23 |
|  |  |  | P4 | 6-9 |
|  |  |  | P5 | 9-11 |
| Pr | Red-brown eyed | 6-18 | P6 | 8-17 |
|  |  |  | P7 | 21-28 |
| Pd | Dark-brown eyed | 36-48 | P8 | 12-19 |
| Pdl | Dark brown eyed,  light thorax  pigmentation | 12-24 | P9 | 10-14 |
|  |  |  | P10 | 6-10 |
| Pdm | Dark brown eyed,  medium thorax  pigmentation | 30-42 | P11 | 7-9 |
|  |  |  | P12 | 8-13 |
| Pdd | Dark brown eyed,  dark thorax  pigmentation | 6-18 | P13 | 6-9 |
|  |  |  | P14 | 16-20 |
|  |  |  | P15 | 12-17 |
|  |  |  | P16 | 5-7 |

**Supplemental Table S2.** Comparison on pupal stages between *A. mellifera* and *B. impatiens* workers. ^a^ *A. mellifera* staging based on Rembold, Kremer & Ulrich (1980); ^b^ Data is represented as 95% confidence interval for medium sized workers

**Rembold H, Kremer J-P, and Ulrich GM. 1980**. Characterization of postembryonic developmental stages of the female castes of the honey bee, *Apis mellifera* L. *Apidologie* **11**:29-38 DOI: doi.org/10.1051/apido:19800104.
